# The Use of Lactide Polymers in Bone Tissue Regeneration in Dentistry—A Systematic Review

**DOI:** 10.3390/jfb14020083

**Published:** 2023-01-31

**Authors:** Justyna Rajewska, Jakub Kowalski, Jacek Matys, Maciej Dobrzyński, Rafal J. Wiglusz

**Affiliations:** 1 Department of Pediatric Dentistry and Preclinical Dentistry, Wroclaw Medical University, Krakowska 26, 50-425 Wroclaw, Poland; 2 Laser Laboratory Dental Surgery Department, Wroclaw Medical University, Krakowska 26, 50-425 Wroclaw, Poland; 3 Department of Orthodontics, Technische Universitat Dresden, 01307 Dresden, Germany; 4 Institute of Low Temperature and Structure Research, Polish Academy of Sciences, Okolna 2, 50-422 Wroclaw, Poland

**Keywords:** augmentation, bone tissue, guided bone regeneration (GBR), bio-ceramics, biopolymers, dental surgery

## Abstract

(1) Background: Different compositions of biodegradable materials are being investigated to successfully replace non-resorbable ones in bone tissue regeneration in dental surgery. The systematic review tried to address the question, “Can biodegradable polymers act as a replacement for conventional materials in dental surgery procedures?” (2) Methods: An electronic search of the PubMed and Scopus databases was conducted in October 2022. The following keywords were used: (lactide polymers) and (hydroxyapatite or fluorapatite) and (dentistry) and (regeneration). Initially, 59 studies were found. Forty-one studies met the inclusion criteria and were included in the review. (3) Results: These usually improved the properties and induced osteogenesis, tissue mineralisation and bone regeneration by inducing osteoblast proliferation. Five studies showed higher induction of osteogenesis in the case of biomaterials, UV-HAp/PLLA, ALBO-OS, bioresorbable raw particulate hydroxyapatite/poly-L-lactide and PLGA/HAp, compared to conventional materials such as titanium. Four studies confirmed improvement in tissue mineralisation with the usage of biomaterials: hydroxyapatite/polylactic acid (HA/PLA) loaded with dog’s dental pulp stem cells (DPSCs), Coll/HAp/PLCL, PDLLA/VACNT-O:nHAp, incorporation of hydroxyapatite and simvastatin. Three studies showed an acceleration in proliferation of osteoblasts for the use of biomaterials with additional factors such as collagen and UV light. (4) Conclusions: Lactide polymers present higher osteointegration and cell proliferation rate than the materials compared. They are superior to non-biodegradable materials in terms of the biocompability, bone remodelling and healing time tests. Moreover, because there is no need of reoperation, as the material automatically degrades, the chance of scars and skin sclerosis is lower. However, more studies involving greater numbers of biomaterial types and mixes need to be performed in order to find a perfect biodegradable material.

## 1. Introduction

Biodegradable materials tend to attract attention from researchers, as the demand for absorbable devices used in the postsurgical osteosynthesis is high [1]. In the treatment of bone defects, scaffolds made of biodegradable materials can provide a platform for cells and growth factors, which will eventually become degraded and absorbed in the body and replaced by the new bone tissue [2]. The high amount of up-to-date scientific literature publications commenting on the topic of biomaterials application in dental surgery which are cited in this study proves that the possibilities of treatment modalities in the sector of maxillofacial surgeries are constantly evolving and researchers do not stop seeking the most efficient solutions. In the last years, many researchers focused on studies on the use of a mix of polylactides with bio-ceramics in bone surgery. These materials are used in procedures such as bone augmentation surgeries prior to the implant placement [3,4,5,6,7,8,9,10,11], sinus lift operations [12,13], orthognathic surgeries [14,15,16], maxillofacial osteotomies [17,18], periodontal disease treatment [19], supplementation of bone defects after tumour removal [20] and treatment of traumas of bones of the masticatory system and facial skeleton [21,22,23]. Depending on the study, the biomaterials are introduced in the form of membranes [19,24], screws [13,14,15,16,17,18,20,21,23,25,26,27], plates [14,15,16,17,18,21,23,26], nanotubes [6], trays [3], distraction devices [7], discs [28], filaments [29] and sheets [22].

Researchers place the biggest emphasis on investigation of the following two novel materials: the first one is a mix of nanohydroxyapatite, polylactide LD in the form of the racemate (LDA) and poly lactic-co-glycolic acid (PLGA) copolymer; the second one is a mix of nano fluorapatite, polylactide LD in the form of the racemate (LDA) and poly lactic-co-glycolic acid (PLGA) copolymer. The research demonstrated that materials of this exact composition have not been a topic of any study regarding regenerative osteosynthesis in the dental field. Nevertheless, the use of nano hydroxyapatite and poly lactic-co-glycolic acid in dental surgery has been noticed within the literature [30,31,32]. Lactic acid is a monomer that can be obtained from natural products, such as corn, sugarcane, or cassava, by the process of sugar fermentation, and, by the latter, condensation transforms into polylactic acid (PLA). The degradation of PLA is based on hydrolysis. It has two steps: firstly, the ester groups of the compound undergo cleaving and reducing the molecular weight, followed by macrophage metabolism and phagocytosis of the lactic acid and oligomers by water and carbon dioxide. PLA exists in the form of stereoisomers, Poly-l-lactide (PLLA), Poly-d-lactide (PLDA) and Poly-DL-lactide (PDLLA), which differ not only by spatial configuration but also by their properties. The L isomer represents a structure of higher crystallinity than the right isomer, which results in higher sheer viscosity and a more gradual rate of resorption [33]. Moreover, PLLA shows an increased modulus of elasticity and tensile strength compared to PDLA. Due to the study of Pawar et al. [34], in 2010, PLA was regarded as the second most important bioplastic worldwide.

PLGA-poly(D, L-lactic-co-glycolic acid) is a copolymer of PLA (polylactic acid) in the form of PDLLA and PGA (polyglycolic acid). Even though PGA is hydrophilic, PLGA is characterized by hydrophobicity. PLGA degrades by similar hydrolysis processes as PLA [35]. However, the higher the concentration of glycolide units to lactic units, the faster the PLGA’s degradation rate, according to the study by Makadia et al. [36]. The PLGA is proven to cause an indigenous inflammation reaction after implantation due to accelerated degradation of the polymer after lowering the local tissue pH due to degradation products. Pandey et al. [37] mention that the FDA has approved PLGA for use in drug delivery devices. The biodegradable synthetic polymers possess hydrophobic surfaces, unprofitable to osteoblasts due to higher apoptosis and lower proliferation rate when compared to hydrophilic surfaces. Due to the polyester’s area being bioinert, the described materials can serve as good osteoconductors but never present osteoinductive features [38].

HAp-hydroxyapatite and FAp-fluorapatite are both bio-ceramics used for biomedical purposes, often applied together with polymers [39]. Calcium phosphate ceramic materials support the regenerative process by their unique properties: perfect biocompatibility and bioactivity, availability due to the ease of synthesis and natural origin of the materials and, most importantly, the hydrophilicity and osteoinductivity. These materials improve osteoblast differentiation and accelerate osteosynthesis, as is richly described in Eliaz et al. [40]. Borkowski et al. [41] in their study mentioned that a highly porous HAp surface causes intense uptake of Ca^2+^ ions from the tissues, decreasing osteoblasts viability and leading to false cytotoxicity. The calcium phosphate ceramics containing fluoride manifest lower porosity and greater density than HAp, having an impact on absorbing smaller amounts of water, calcium and phosphate ions but releasing great but non-toxic amounts of fluoride. The combination of bioplastic and bio-ceramic materials used as postsurgical scaffolds promoting healing has been a topic of many studies, which this study aims to collect, compare and analyse [42].

Despite the growing popularity of alloplastic materials used in osteoregeneration, no systematic reviews have yet been published concerning the use of different kinds of polylactides in various types of dental surgeries. Novel treatment modalities develop daily, followed by the development of modern materials. This study thoroughly analyses accessible publications and sums up current knowledge about the use of lactide polymers with hydroxyapatite or fluoroapatite in bone tissue regeneration, providing clinicists with the most up-to-date information about these biomaterials.

## 2. Materials and Methods

### 2.1. Focused Question

The focused question in the review was: “Can biodegradable polymers act as a replacement for conventional materials in dental surgery procedures?”

### 2.2. Protocol

**Figure 1:**
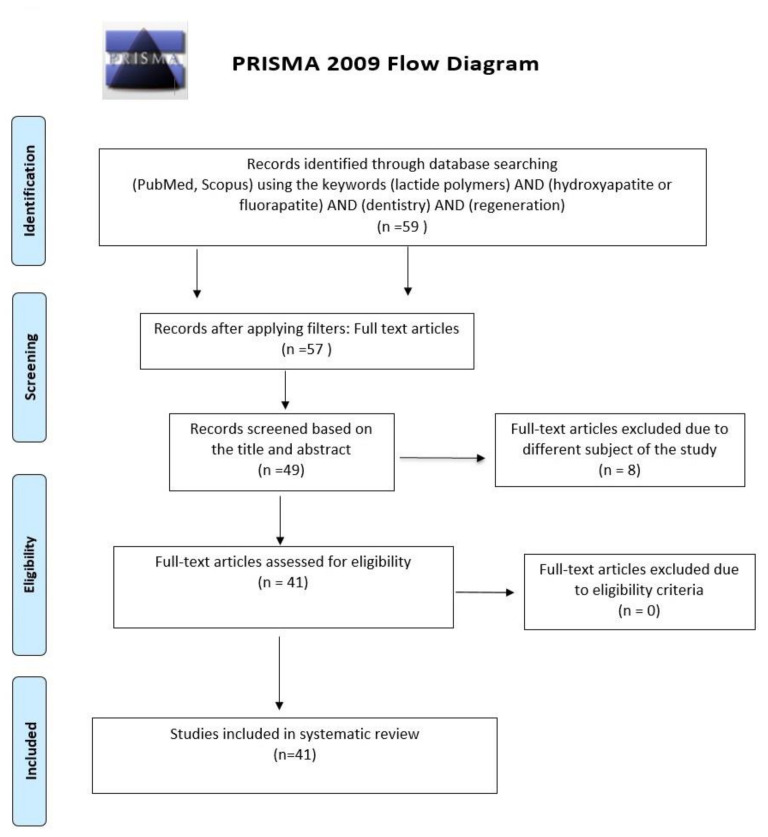
A detailed selection of the articles in the review was constructed according to the PRISMA 2009 Flow Diagram.

The review was scheduled per the PRISMA statement [43] and the *Cochrane Handbook of Systematic Reviews of Interventions* [44]. Details of the assignment criteria are presented in Figure 1.

### 2.3. Eligibility Criteria

Only studies which met the following criteria were included in the review:

Inclusion Criteria:

- In vitro and in vivo (human and animals) studies
- Studies in which the material used was based on a combination of synthetic lactide polymers and bio-ceramics
- Studies in which the material used was based on a combination of synthetic lactide polymers and bio-ceramics and an additional material/factor
- Studies that obtained a clear result on whether the used materials do or do not influence bone regeneration processes
- Studies whose goal was an assessment of the material mix, not a single material
- Studies that examined the material itself, not the properties of an ingredient added
- Articles were written at any time by any research group but only in the English language
- Research included in vivo studies performed on human or animal bodies not only in the field of dentistry in order to study the materials’ behavioural traits and clinical properties in as broad a perspective as possible

Exclusion criteria:

- Non-English papers;
- Opinions;
- Letters to the editor;
- Editorial papers;
- Review articles;
- Clinical reports;
- No full-text accessible;
- Duplicated publications.

### 2.4. Information Sources, Search Strategy and Study Selection Process

A detailed literature review in PubMed and Scopus databases was conducted in October 2022 to obtain articles covering osteosynthesis achieved using a material mix of bio-ceramics (hydroxyapatite or fluorapatite) and polylactide (PDLLA and PLGA). To achieve proper and filtered search results, the terms (lactide polymers) AND (hydroxyapatite or fluorapatite) AND (dentistry) were applied. The reviewers restricted the trawl to studies meeting the eligibility criteria. Both in vivo studies performed on humans and animals, as well as in vitro laboratory studies, were included in the research. In vivo studies included procedures associated with dentistry and surgeries and examinations performed on other parts of human/animal bodies. Including that parameter gave reviewers a better insight into the tested materials’ behavioural traits and clinical properties. Reviewers decided to include studies describing the mix of bio-ceramics with other kindred polylactides (such as PLCL), apart from PDLLA and PLGA, in order to scan and compare their application in the osteosynthesis with materials being the focus of the study. Moreover, the studies in which another additional substance, apart from bio-ceramics and polylactides, had been used to fabricate the material were also included. Instead of evaluating the osteogenic properties of bio-ceramics mixed with polylactide, articles assessed the properties of an additional material affixed to the mix, and its impact on the tissue or original materials mix have not been included. However, studies in which another material was added to the mix and various tests were performed to assess its osteogenic properties were counted as relevant. Studies that were not available in a full-text form and those written in a different language than English were excluded and are presented in the table “Excluded Studies”. The researchers did not find any systematic reviews related to this topic (see Table 1).

### 2.5. Data Collection Process and Data Items

Data extracted from the studies were collected by two researchers independently. The following information was collected: name and authors of the article, the main material used in the study, material used for comparison (if applicable), form of the applied material, type and percentage volume of hydroxyapatite fluorapatite constituting of an applied material, type of study (in vivo on humans, in vivo on animals, in vitro), place of the insertion of the material in human/animal body (if applicable), type of surgery performed on the subject (if applicable), period of time until check-up and testing, in addition to the method of check-up and testing, the aim of the study and results obtained. No automation tools were used in the process. Collected data were used to create a table in Microsoft Excel.

### 2.6. Risk of Bias

In order to minimize the risk of bias, two researchers working independently examined the studies by their abstract, as well as by the full text if needed. To establish the degree of agreement, the Cohen’s kappa equation was implemented. Any variance of appropriateness and inappropriateness of the study was discussed by the authors. The scores of each study were calculated, and an overall evaluated risk of bias (low, moderate or high) was made for each included study, as suggested in the *Cochrane Handbook for Systematic Reviews of Interventions* [63].

### 2.7. Effect Measures and Synthesis Methods

The data collected from articles were used to create a table. To tabulate results thoroughly and adequately, researchers needed to gain access to the full-text documents. Most of the results obtained from the research cannot be displayed by means of a numerical presentation and are presented in the form of texts. The statistical data describing the intra-individual mean values of the percentage content of a HApHAp/FApFAp in each study were calculated by the researchers. Study nr [64] did not provide clear data on the precise content of the material. Studies nr [11,27,65,66,67,68] did not supply enough information to calculate the material’s content in the final material mix. The researchers performed the calculations of quantities and statistics of subjects in in vivo studies. Study nr [28] and study nr [26] did not define the number of subjects; therefore, those studies were not used for precise quantitative measurements. The mean values and standard deviations of age shown in vivo studies column of the table are extracted from the original texts. The researchers prepared statistical evidence of methods and periods until diagnosis in vivo studies. Among 28 in vivo studies, only one of them [26] did not define the period until reexamination.

### 2.8. Quality Assessment

Two researchers analysed the studies independently in order to assess their quality value. To evaluate each study, specific parameters were used, each being graded individually. The criteria and scoring system are as follows: (1) material used in the study: a score of three points was given if the material was a mix of HAp/FAp and PLGA or PDLLA since these materials are the true point of the study; a score of two points was given if the material was a mix of HAp/FAp and any polylactide other than PLGA or PDLLA since it provides similar results and can act as a basis for future research; a score of one point was given if the material was a mix of HAp/FAp and a polylactide with another supplementary substance or produced under the influence of an additional factor. (2) Comparative material or a control test: one point for comparison with a different material or with a control sample; zero points for no material compared. (3) Content of HAp/FAp in the material expressed by the percentage value: one point given for precise information about content; zero points given for no information about content; “ns” stands for “not specified” and was treated as zero points, given for unclear or incomplete information about the content. Reviewers agreed that these three factors were the essential variables in each study. This information gave each study the most crucial data on the materials’ properties and behaviour in different conditions and environments. They were sufficient to assess the accessibility, draw conclusions and articulate the results. Therefore, the minimum point value possible to obtain was one, and the maximum value was five. The higher the score, the more qualitative the study and the more applicable the data presented in the studies.

## 3. Results

### 3.1. Study Selection

A total of 59 articles have been initially screened for applicability in this systematic review. Two of the publications have not been found with a full text available. After analysing titles and abstracts, a total of eight articles were excluded due to other objects of study (studies not concerning bone regeneration; studies describing effects of additional substances on scaffold materials). The full-text examination helped to reject eight more studies in which inclusion criteria were not met (other studies’ subject— biomaterials used for regenerative bone examinations—were different from the material screened initially). Cumulatively, 41 studies were included in the qualitative synthesis.

### 3.2. Study Characteristic

Forty-one studies were included in this review. Each of them has been thoroughly assayed and screened to obtain data useful for a general comparison. Specific parameters of the studies were tabulated in four complementary tables, further divided into parts (see Table 2: in vivo examination of materials—a mix of polylactide and bio-ceramics, composed of 21 studies; Table 3: in vivo examination of materials—a mix of polylactide, bio-ceramics and an additional factor or material, composed of 6 studies; Table 4: in vitro examination of materials—a mix of polylactide and bio-ceramics only, composed of 5 studies; Table 5: in vitro examination of materials which were a mix of polylactide, bio-ceramics and an additional factor, composed of 9 studies). The subject of included studies was a material mix of bio-ceramics with polylactides. Polylactides in tabulated records are PLGA, PLA, PLA + PGA, PLLA and PLCL. The most popular choice of material to compare with was titanium. Some studies compared a controlled trial in the form of spontaneous healing instead of providing material to compare. Almost half of the studies delivered information on the percentage content of HAp in the material, and many studies described a form of its application. Researchers decided to include both in vivo human and animal as well as in vitro studies in the review. To obtain even more specific data, in vivo studies provided information about the type and number of examined species, the type of surgery performed on them, the place of material implantation, and periods and methods of postoperative reevaluation (see Table 2, Table 3, Table 4 and Table 5).

### 3.3. Subjects of the Study

There is heterogeneity in the papers regarding composition and form of application. All the studies in the review concerned composites of hydroxyapatite (HAp) particles and poly (L-lactide) (PLLA), which were compared to different materials in terms of quality and properties. The studies can be divided based on the other materials compared to the primary bioresorbable material (HAp/PLLA) to show the main result, proving HAp/PLLA to be a valuable and sufficient alternative. The tested material was applied in different types that included screws, mini-screws, scaffolds, 3D mesh trays, plates, filaments, polymer discs, nanotubes and composite sheets. The percentage of HAp/FAp in the examined material also varied; it was mainly 30% for the screws and 40% plates, but also 1%, 4,5%, 5%, 10%, 20%, 25%, 40%, 50%, 66% and 70%, or was not given. In the following studies, the biomaterial had comparable properties to the conventional ones. The study of Murat Cavit Cehreli et al. [64] proved that the stability of the bioresorbable (u-HA/PLLA) mini-plates and screws based on hydroxyapatite and polylactide are comparable to titanium ones. Studies of Koichiro Ueki et al. [18], K. Ueki, et al. [15] and Ueki Koichiro et al. [16] assessed bone healing after Le Fort I osteotomy with the use of uHAp/PLLA, titanium and PLLA, showing no crucial differences in bone defects among the plate types. Studies where HAp/ PLLA achieved better results than titanium included Y. Shikinami et al. [26], Akihiro Takayama et al. [13], Akira Matsuo et al. [3], C Amnael Orozco-Díaz et al. [29], J.M. Taboas et al. [70] and Akira Matsuo et al. [20]. In the study of Ueki Koichiro et al. [26], raw HAp/PLLA in the form of mini-screws and implants was compared to titanium and raw PLLA, where it exhibited significantly better results after inspection of fixation strength. In the study of Akihiro Takayama et al. [13], uHAp/PLLA and UV-HAp/PLLA traded with ultraviolet light were used in the form of absorbable screws and compared to titanium. In vitro studies reported that uHAp/PLLA exposure to UV changed the material’s properties from hydrophobic to hydrophilic, allowed uHAp to become exposed, improved osteoconductivity and surface contact, induced osteoblasts differentiation and increased the number of attached bone marrow cells. The study of Bryan Taekyung Jung et al. [69] compared hydroxyapatite/poly(L-lactide) (HAp-PLLA) and titanium (Ti), magnesium alloy (Mg alloy), poly-L-lactic acid (PLLA) used for fixation of subcondylar fractures considering stress distribution. HAp-PLLA showed less stress distribution on the non-fractured side in comparison with PLLA, but the values were still more significant than those of non-biodegradable devices. In the study of Kyung Mi Woo et al. [71], it was proved that by adding HAp into a biopolymer scaffold, cells were protected from undergoing apoptosis by the adsorption of serum proteins. Implants made of HAp were substantiated to absorb more serum fibronectin and vitronectin and bind more purified interns than titanium implants. In the study of Akira Matsuo et al. [20], it was proved that better bone formation is obtained with PLLA/HAp screws than with titanium ones due to higher CT value of the PCBM and PRP. Additionally, in some studies, the bio-material was combined with supplementary factor/component and then compared to conventional materials such as collagen, simvastatin, dental pulp stem cells and calcium phosphate salts (HAp/β-TCP), mediated with HAS surfactant, loaded with Dexamethasone, metronidazole, platelet-derived growth factors, B-cyclodextrin grafted nano-HAp + simvastatin, poly-maleate (4PLAUMA) elastomer with nHAp, BMP-2 (bone morphogenetic protein 2) + b TCP cryogen composite, all described in Table 1 and Table 2, “examining materials being a mix of polylactide, bio-ceramics and an additional factor or material”. In some studies, HAp/PLLA was not compared to any other material but tested alone, showing valuable properties. These include the studies of Shintaro Sukegawa et al. [25], Sun Jae Lee et al. [23], Jung Bok Lee et al. [76] and Jung Hyun Park et al. [17]. In the study of Shintaro Sukegawa et al. [25], uncalcined and unsintered HAp with PLLA was applied in the form of screws and used together with a bone graft to obtain a proper bed for dental implants. On the histopathological examination, the new bone, containing osteocytes, osteoblasts and lamellae, was mixed and connected with the biomaterial. The immunohistochemical analysis revealed the presence of CD68 antigen. An immunohistochemical analysis made it clear that the novel material has osteogenic properties by evaluation of the presence of preosteoblasts-, Osterix-, RUNX2- and mSOX9-. The research confirmed the biodegradable and osteoconductive properties of u-HAp/PLLA. In the study of Sun Jae Lee et al. [23], surgical treatment of mandible fracture was performed using an unsintered Hydroxyapatite/Poly(L-Lactide) Composite Fixation System. The size of u-HAp particles, which are crushed to 3–5 mm diameter, allow the phagocytosis to occur and bond to the PLLA matrix, resulting in high bioactivity. In the study of Adil Akkouch et al. [68], simvastatin was incorporated into the fibrous and cylindrical structure of PLLA with HAp, which resulted in releasing and loading simvastatin and osteoblast responses-stimulation of bone formation.

### 3.4. Main Study Outcomes

The studies included in the review varied with the type of conduction, material compared and form of applied material. Type of conduction: in vivo [3,4,5,6,7,8,9,10,11,12,14,15,16,17,18,20,21,22,23,25,26,27,28,64,65,66] or in vitro [19,24,29,67,68,69,70,71,72,73,74,75,76,77], type of surgery, and whether there was a material compared [3,6,8,9,10,11,13,14,15,16,18,21,24,26,27,28,29,64,65,66,67,68,69,70,71,72,73,74,75,77] or not [4,5,7,17,19,20,22,23,25,76]. All the studies included in the review concerned composites of hydroxyapatite (HA) particles and poly(L-lactide) (PLLA) in different forms: almost always raw, forged, uncalcined and unsintered, sintered [70], traded with UV light [13], with additional factors such as simvastatin [10,65,76], collagen [67,68] and differently shaped membranes [24,77], screws [13,14,15,16,17,18,20,21,23,25,26,27], plates [14,15,16,17,18,21,23,26], nanotubes [6], trays [3], distraction devices [7], discs [28], filaments [29], sheets [22] and others. The researchers decided to have a more general look at assessing the clinically valid properties of chosen materials and their use in bone regeneration. The primary study outcome is that despite differences in the form of applied material and type of surgery, the bioresorbable materials possess good biocompatibility [4,5,29] osteoinductive properties [3,4,13,19,25] and other clinically valid properties benefitting bone regeneration procedures. This could be concluded by observing faster new bone formation [7,9,11,13,20,64,76], good bone quality postsurgically [3], no inflammation in situ [5,25,26,64], lower immune response [9], no sign of infection [22,23,66], suppression of apoptosis [71], visible neoangiogenesis [5,9], more serum fibronectin and vitronectin absorption [71], increased growth factor production and protein absorption [71] and no cytotoxicity in situ [4,6]. No bioresorbable materials mentioned in this review caused cytotoxicity, which is an advantage of the usage of biopolymers. The elaborate explanation of the importance of cytotoxicity and the specific particle interactions in biological environment is described in the study of Olcay Özdemiret et al. [78]. In the study of Marco C. Bottino [19], the bioresorbable material is considered functional, with osteoconductive, inductive and antibacterial properties which cause no cytotoxicity. In the study of Murat Cavit Cehreli et al. [64], a chemically-synthesized poly(L-lactide)- hydroxyapatite (PLLA–HAp) composite was used and no inflammation processes were detected; there were only some HAp particles which did not completely degrade, surrounded totally by bone cells or connective tissues, which did not cause any cytotoxic reactions. The advantages of assimilation of the biodegradable materials to metallic ones are also lack of scars and skin sclerosis due to reoperation and the need for removal of the metallic material from the tissue, no risk of rejection of the material and undetectability of the device after its full utilization [26]. Moreover, HAp/PLLA composites can be applied as thinner planes possessing the same elastic modulus, allowing faster resorption and lower palpability than metallic ones [26,73] (see Table 2, Table 3, Table 4 and Table 5).

### 3.5. Quality Assessment

In total, 1 study obtained 5 qualitative points (low risk of bias), 11 studies obtained 4 qualitative points (low risk of bias), 17 studies obtained 3 qualitative points (moderate risk of bias) and 13 studies obtained 2 qualitative points (high risk of bias), shown in Table 6.

## 4. Discussion

Bioresorbable composites of HAp/PLLA were proven to be useful, possess clinically valid properties, and to be capable of tissue regeneration in surgical procedures. Most studies that met the inclusion criteria and were considered in the review showed that biodegradable polymers might replace conventional materials in dental surgery procedures as they have equal or superior properties. The focus was put on the materials, which are a mix of polylactide and bio-ceramics only or bio-ceramics and an additional material or factor. The other materials usually improved the properties and induced osteogenesis, tissue mineralisation and bone regeneration by inducing osteoblast proliferation.

This review systematically assessed the impact of used materials on osteogenesis that was altered either by a composition of the material or additional factor. A factor that improved the osteogenic properties of bioresorbable material was UV light, used in the study of Miguel Noronha Oliveira et al. [65]. Results proved that specimens with UV-HAp/PLLA presented the highest ratio of new bone, followed by Ti and uHAp/PLLA presenting the lowest. UV-exposed material has been proven to present the best osteogenic properties because of the early differentiation of preosteoblasts and promotion of the adhesion of blood or cells. The study tested and compared a record number of 20 biological properties of ALBO-OS with Geistlich Bio-Oss^®^. ALBO-OS represents a 4.5 times higher solubility range, resulting in faster new bone formation. Another study proving osteogenic properties of bioresorbable material was the study of Akira Matsuo et al. [3], where a custom-made bioresorbable raw particulate hydroxyapatite/poly-L-lactide mesh tray with particulate cellular bone and marrow and platelet-rich plasma was compared with titanium trays. Bone conduction and induction properties turned out to be higher in the graft fabricated from PCBM rather than a block of bone. The same result was obtained for PLGA/HAp mix in the study of J.M. Taboas et al. [70]. Implants made of HAp were substantiated to absorb more serum fibronectin than titanium implants. 

Another variable considered is tissue mineralization; some studies showed that biomaterials with additional factors tend to increase it. The study of Jung Bok Lee et al. [10] proved that the incorporation of hydroxyapatite and simvastatin into the material enhanced mineralization and ALP activity. Authors agreed that the scaffolds possess a proper microenvironment for differentiation and growth of human adipose-derived stem cells. In the study of Rung-Shu Chen et al. [66], the group of material with DPSCs showed higher mineralization tissue number and volume fraction as well as structure thickness. In the study of Adil Akkouch et al. [68], the primary material tested Coll/HAp/PLCL provided better adhesion to DPSCs, DPSCs grew faster in the mixed material, alkaline phosphatase activity was higher and grew more rapidly in the mixed material and tissue mineralization was higher. In the study of Idalia A. W. Brito Siqueira et al. [6], VACNT-O:nHA increased the crystallization rate in PDDLA material. PDLLA/VACNT-O:nHAp caused higher carbonated peaks compared to PDLLA. All scaffolds induced mineralisation and no cytotoxic effects were present. 

Biomaterials with additional factors tested in the studies proved to be able to promote osteoblast proliferation. In the study of Ahmed Talal et al. [24], the percentage of the material in composites affected its properties. The lowest percentage material had the highest osteoblasts proliferation rate. High concentration material had the highest ALP activity and was stated as a useful material for application of a GTR membrane. In the study of Adil Akkouch et al. [67], scaffolds were made of mineralized type I collagen (Coll), hydroxyapatite (HA), and poly(lactide-co-e-caprolactone) (PLCL) and cultured before testing. Thermal and mechanical evaluations proved that the material is a resistant and elastic scaffold, able to promote osteoblast adhesion and proliferation. In the study of Akihiro Takayama et al. [13], uHAp/PLLA exposure to UV changed the properties of material from hydrophobic to hydrophilic, allowed uHAp to become exposed, improved osteoconductivity and surface contact, induced osteoblasts differentiation and increased the number of attached bone marrow cells. In turn, a systematic review published by Anne Handrini Dewi et al. [79] described the use of hydroxyapatite in chosen dental surgeries. The study analysed the effects of hydroxyapatite-based materials mixed with autografts, allografts, xenografts and alloplastic grafts, such as PLGA, on the alveolar bone regeneration, asserting the autograft as a golden standard. The study mentioned that PLGA/HAp has a potential for sinus lift augmentation; however, the reconstructed bone had insufficient quantity and quality to insert endosseous implants. More research needs to be done and a longer observation period for more accurate results and confirmation of findings.

## 5. Conclusions

From the included studies, it can be concluded that materials of polylactide and bio-ceramics, whether alone or in a mix with an additional factor, can be sufficient or superior to conventional materials like titanium. Biomaterials were tested and showed better osteoinductive properties, promoted cells proliferation (ex. PDGF), decreased the time of apatite layer formation and improved antibacterial properties (metronidazole). It was also proven that increased amounts of HAp decreased the degradation rate of the material. The studies showed the advantages of assimilation of the biodegradable materials to metallic ones: lack of scars and skin sclerosis due to reoperation and the need for removal of the metallic material from the tissue, no risk of rejection of the material and undetectability of the device after its full utilisation. Biomaterials also showed advantages in aspects of biocompatibility, bone remodelling and healing time. The studies included in the review proved that biodegradable polymers could be successfully used instead of conventional materials, depending on the properties that are needed in a given case. Lactide polymers might play a significant role in the future of bone regeneration due to the ease, cheapness and ethics of its obtainment as well as the possibility of machining its production. The perfect biodegradable material has not yet been found; therefore, clinicians, bioengineers and researchers should not stop the search. In the future, a greater systematic review should be performed, inspecting more kinds of biomaterials than the polylactides and bio-ceramics presented in this study.

## Figures and Tables

**Table 1:** Excluded Studies.

Ordinal Number	Reason for the Exclusion of the Study	Title and Author
1	Different object of the study	Cijun Shuai [45]
2	Different object of the study	S Kono [46]
3	Different object of the study	Ming Bi [47]
4	Different object of the study	Jun Makiishi [48]
5	Different object of the study	Wei Fan [49]
6	Different object of the study	Uwe Gbureck [50]
7	Different object of the study	Fang Mei [51]
8	Different object of the study	Shokoufeh Shahrabi-Farahani [52]
9	Different subject of the study	Ayse Sumeyye Akay [53]
10	Different subject of the study	Yevgeny Sheftel [54]
11	Different subject of the study	Cigdem Atalayin [55]
12	Different subject of the study	Vineet Kini [56]
13	Different subject of the study	Masaaki Takechi [57]
14	Different subject of the study	H Schliephake [58]
15	Different subject of the study	Florian G Draenert [59]
16	Different subject of the study	Mona K Marei [60]
17	No full text	A Ashman [61]
18	No full text	O Skochylo [62]

**Table 2:** In vivo examination of materials—a mix of polylactide and bio-ceramics.

Reference	Examined Material	Material Compared	Form of Applied Material	Type and Percentage of HAp/HAp in the Examined Material	Examined Species	Type of Surgery Performed	Results Short
Akiro Matsuo [20]	(HAp)/ poly-l-lactide (PLLA)	No material compared	8 × 2.0 mm Screws	Unsintered, uncalcined, 40 wt.% HAp	In vivo; 29-year-old woman, 66-year-old man	Ostheosynthesis after resection of tumors	Higher osteogenesis in PLLA/HAp sample than titanium.
Akira Matsuo [3]	HAp/PLLA	Titanium trays	Mesh trays	Unsintered and uncalcined 40% HAp	In vivo 14 beagle dogs	Implantation in mandible	The bone quality of 2 samples similar after 12 months, the bone remodeling on the resorbable sample delayed by 6 months. The novel material well adapted to the mandible.
Andrea Vaz Braga Pintor [4]	PLGA (Poly(lactide-co-glycolide)/nanoescale hydroxyapatite (ReOss^®^, Intra-Lock International)	Healing without material	Powder and putty configurations of composite	50% wt.	In vivo 18 white New Zealand rabbits	Implantation in calvaria	Biocompability of both material forms is similar. Clinical applicability of the two forms is different.
Constantin A. Landes [14]	HAp/PLLA (forged unsin- tered hydroxyapatite e Poly L-lactide; Osteotrans MX, Takiron, Osaka, Japan)	Titanium miniplates	Plates and screws	Not mentioned	In vivo 50 people	Orthognathic surgery	Osteoconductive material was successfully used in orthognathic surgery; however, small, irrelevant relapses were present.
Hideo Shimizu [5]	poly-L-lactic acid (PLLA) and hydroxyapatite (HAp)	No material compared	Nanoparticle composite	1% HAp-1% PLLA and 2% HAp-2% PLLA	In vivo 20 rats	Implantation in calvaria and tibia	Material in both cases presented proper biocompability. In tibia osteogenesis was noted, due to a bone and a defect type.
Idalia A. W. Brito Siqueira [6]	PDLLA/superhydrophilic vertically aligned carbon nanotubes:nanohydroxyapatite (PDLLA/VACNT-O:nHAp) scaffolds	PDLLA as control, PDLLA/VACNT-O:nHAp1, and PDLLA/VACNT-O:nHAp2	Nanotubes	Not mentioned	In vivo adult male mice (22–28 g) and in vitro	Implantation in calvaria	A scaffold induced bioactivity did not present any cytotoxicity and promoted bone remodeling. It yielded better propertied than PDLLA alone.
In-Seok Song [21]	unsintered hydroxyapatite particles and poly-L-lactide (u-HAp/PLLA)	Titanium	Miniplates and screws	Unsintered, miniplates 40% wt HAp, screws 30% wt HAp	In vivo 40 people (12 females, 28 males).	Mandibular body fracture fixation	u-HA/PLLA miniplates and screws presented comparable stability to titanium ones, even though some displacements were observed.
Jung Hyun Parl [17]	hydroxyapatite/poly-L-lactide; Osteotrans MX, Takiron, Osaka, Japan	No material compared	Plates and screws	Not mentioned	In vivo 53 patients	Le Fort I osteotomy	Vertical relapses were present in the posterior maxilla, depending on the range of surgical movement of the bone. Clinically acceptable.
Koichiro Ueki [18]	uncalcined and unsintered hydroxyapatite and poly-L-lactic acid (u-HAp/PLLA)	Titanium, PLLA	Plates and screws	Uncalcined, unsintered	In vivo 18 Japanese adults	Le Fort I osteotomy	The healing was not completed in 1 year span after incorporation of the absorbable material. However, the areas of bone defects were smaller.
K. Ueki [15]	unsintered hydroxyapatite (u-HAp)/poly-L-lactic acid (PLLA)	PLLA, titanium	Mini plates with screw	uncalcined and unsintered hydroxyapatite (u-HA), 30 wt.% screw, 40 wt.% plate	In vivo 60 Japanese adults	Orthognathic surgery, Le Fort I with SSRO	No major differences between 3 different materials were discovered after the treatment.
Murat Cavit Cehreli [64]	chemically-synthesized poly(L-lactide)–hydroxyapatite (PLLA–HAp) composite	Healing without any material	Scaffold	Not specified	In vivo 4 dogs	Ridge preservation after premolars loss	Both groups revealed similar results in bone healing. PLLA-HA proved that it can be applied in maxillofacial structures treatment.
Osama Zakaria [7]	poly-L-lactide/hydroxyapatite	Nothing	Distraction device	40 wt.%	In vivo 8 male white Japanese rabbits	Implantation in calvaria	Periosteal distraction appliance was discovered to be of potential use in vertical augmentation procedures of maxillofacial structures. The optimal distraction range is 330 μm per day or less.
Ruggero Rodriguez y Baena [12]	poly(lactic-co-glycolic) acid/hydroxyapatite (PLGA/HAp)	deproteinized bovine bone (DBB)	Not mentioned	Ca-HAp	In vivo 8 patients	Sinus lift	According to the study, it is sufficient to use PLGA/HAp in the sinus-lift surgeries; however, DBB graft provides better bone healing.
Shintaro Sukegawa [25]	Uncalcined and unsintered HAp with PLLA	No other material	Screws	Uncalcined and unsintered HAp, 30 weight fractions	In vivo 5 patients	fixation of mandibular ramus bone graft used for alveolar ridge augmentation	HAp/PLLA showed prime results in the studies, and it was proven to act as a substitute in procedures requiring osteosynthesis.
Shinya Tsumiyama [22]	u-HAp/PLLA	No material compared	Smooth composite sheet with no holes	Unsintered	In vivo 72 patients	Reconstruction after orbital fracture	The study proved applicability and safety of u-HA/PLLA usage in orbital wall fractures reconstruction.
Sun Jae Lee [23]	Unsintered HAp/PLLA	No material compared	Plates and screws	Unsintered	In vivo 13 people	Mandibular fracture fixation	The study proved that u-HAp/PLLA plates can be successfully used in fixation of mandibular fractures and their biggest advantage is absorbability.
T Zislis [28]	PLA-PGA copolymer with hydroxyapatite (HAp)	Plain 50:50 PLA-PGA copolymer, PLA-PGA copolymer with autolyzed antigen-extracted (AA) bone particles	Polymer discs	Not mentioned	In vivo rats	N/a	Study claims that the incorporation of hydroxyapatite accelerates the PLA-PGA copolymer degradation.
Tohru Hayakawa [8]	2 materials: 1. poly(lac- tide-co-glycolide) (PLGA) and Ca-deficient hydroxyapatite (CDHA) 2.poly(lac- tide-co-glycolide) (PLGA) and a mixture of carbonated hydroxyapatite (CHA) and CDHA	PLGA	Composite scaffolds	30% wt	9 Japanese white rabbits	Implantation in tibia	Study proved that the level of crystallinity affects bone response, with the low-level crystallinity material being superior and having great properties and potential use in bone tissue engineering procedures.
Ueki Koichiro [16]	poly-L-lactic acid (PLLA)	PLLA, Titanium	Mini plates and 4 screws	Uncalcined, unsintered, screw 30 wt.% HAp, plate 40 wt.%HAp	In vivo 60 Japanese patients	Bilateral SSRO, orthognathic surgery	No significant differences were found post surgically in time-course changes between all 3 materials.
Vukoman Jokanović [9]	porous calcium hydroxyapatite scaffold covered with poly (lactide-co-glycolide) (PLGA), described as ALBO-OS	Geistlich Bio-Oss^®^ as positive controls and empty defects as negative controls	Not mentioned	Not mentioned	20 rabbits	Implantation in calvaria	According to the study, ALBO-OSS presents great properties and can be safely used in patients requiring maxillofacial or orthopedic surgeries.
Y. Shikinami [26]	Raw hydroxyapatite and poly l-lactide	Raw PLLA; titanium	Mini screws mini plates	30% for mini screws, 40% for manipulates	In vivo beagle dogs	Orthopedic, oral-maxillofacial, craniofacial	Comparison of the HA/PLLA materials with titanium and PLLA in maxillofacial, cranial and oral surgeries proved that the novel material is safer and less objectionable.

**Table 3:** In vivo examination of materials—a mix of polylactide and bio-ceramics with additional material or factor.

Reference Number	Examined Material	Material Compared	Form of Applied Material	Type and Percentage of HAp/HAp in the Examined Material	Examined Species	Type of Surgery Performed	Results Short
Akihiro Takayama [13]	uHAp/PLLA and UV-uHAp/PLLA traded with ultraviolet light	Titanium	Screws	30% raw unsintered HAp	In vivo, 30 rabbits	Sinus lift	In vitro studies reported that uHAp/PLLA exposure to UV changed the properties of material from hydrophobic to hydrophilic, allowed uHAp to become exposed, improved osteoconductivity and surface contact, induced osteoblasts differentiation and increased the number of attached bone marrow cells. Specimens with UV-uHAp/PLLA presented the highest ratio of new bone.
Hao-Chieh Chang [27]	poly(D,L-lactide-co-glycolide) (PLGA) microspheres encapsulating bone morphoge- netic protein-2 (BMP-2) within a gelatin/hydroxyapatite/b-tricalcium phosphate (gelatin/ HA/b-TCP) cryogen composite	gelatin-HA/b-TCP cryogel composite alone (HAP); cryogel composite infused with BMP-2 (BMPi); no cryogel composite (control)	2.5 mm × 5 mm scaffold fixed with a 6mm long and 1.2 mm high titanium screw.	Not specified	In vivo, 16 rats	Alveolar ridge augmentation/preservation	All materials containing gelatin/HA/b-TCP obtained higher relative bone volume than the composite sample. The material composed of PLGA obtained the highest ratio of new bone deposition after the examination period.
Jung Bok Lee [10]	poly(l-lactic acid) (PLLA)/gelatin (PG) fibrous scaffolds, coated onto with β-cyclodextrin (βCD) grafted nano- hydroxyapatite (HAp) via an interaction between βCD loaded onto with Simvastatin (SIM) and adamantane.	Comparison with PGA, PGA-H, PG-H, PG-HB, PGA-HB, HAp	Scaffold	30% wt incorporated in the scaffold, 17% wt present on the coating.	In vivo, 4 male New Zealand rabbit	Implantation in calvaria	The studies proved that the incorporation of hydroxyapatite and simvastatin increased osteodifferentiation of human adipose-derived stem cells as well as growth on the fibrous scaffold, mineralization and ALP activity.
Miguel Noronha Oliveira [65]	poly(D,L-lactide-co- glycolide) with hydroxyapatite/ b-TCP scaffold, (PLGA/ HAp/b-TCP) and PLGA/HAp/b-TCP with 2.0% simvastatin scaffold (PLGA/HA/S),	deproteinized bovine bone mineral with 10% collagen (DBBM-C), spontaneous healing (control)	Scaffold	Not specified	In vivo, 13 patients	Ridge preservation after maxillary wisdom tooth loss	The use of simvastatin did not result in any significant beneficial effects. The study stated that, for future use, materials with higher porosity than between 81% and 91% should be used.
Mohamed H.Helal [11]	CAD-CAMefabricated polylactic acid (PLA) scaffold enriched with calcium phosphate salts including hydroxyapatite (HAp) and beta tricalcium phosphate (b-TCP)	PLA scaffolds	Scaffold designed by CAD CAM to properly fit the bony defect	Not specified	In vivo, 28 beagle dogs	Implantation in mandible	The scaffolds containing calcium phosphate and HAp presented higher new bone formation penetrating in the scaffold.
Rung-Shu Chen [66]	hydroxyapatite/polylactic acid (HAp/PLA) loaded with dog’s dental pulp stem cells	hydroxyapatite/polylactic acid (HAp/PLA) and control group (no material used)	3D printed scaffolds of cylindrical shape	Not specified	In vivo, 2 adult dogs	The materials covered with dental pulp stem cells were inserted into the bone defect in the region of I3 on the left; the materials not covered with dental pulp stem cells were inserted into the bone defect in the region of I3 on the right, the defects in post extraction areas of P2 and P4 were left as control groups. Alveolar ridge preservation/augmentation	The group of material with DPSCs showed higher mineralization tissue number and volume fraction as well as structure thickness.

**Table 4:** In vitro examination of materials-a mix of polylactide and bio-ceramics.

Reference Number	Examined Material	Material Compared	Form of Applied Material	Type and Percentage of HAp/HAp in the Examined Material	Results Short
Bryan Taekyung Jung [69]	hydroxyapatite/poly-L-lactide (HAp-PLLA)	titanium (Ti), magnesium alloy (Mg alloy), poly-L-lactic acid (PLLA)	Mini-plate and screw for each type	Unsintered	Biodegradable materials possess higher values of stress distribution and post surgical deformation than metal materials. The values are not large enough to be important clinically.
C Amnael Orozco-Díaz [29]	polylactic-acid hydroxyapatite (PLA-HAp)	Hip cancellous bone autograft, annealed Pla, pure Pla	a filament manufactured on a 3D printer	5%, 10%, 20%	The novel material based on PLA-HAp proved to be valuable clinically in in vitro studies, especially in the 10% wt form.
J.M. Taboas [70]	PLA/HAp with global pores of diameter 500 um and 600 um	PLA/PGA with global pores of diameter 800 um, PLA with 500 um global pores	Sintered	Not mentioned	Study proved the quality and safety of biodegradable fabrication methods
Kyung Mi Woo [71]	poly(L-lactic acid)/hydroxyapatite (PLLA/HAp)	PLLA	Composite scaffolds	Micro and nano sized HAp	Study proved that the apoptosis rate is lower, and the osteoblasts survival rate is higher in the PLLA/HAp scaffold than in the clear PLLA one.
R.L. Simpson [72]	poly(L-lactide-co-glycolide) (PLGA) semi- crystalline poly(α-hydroxyester) co-polymer with sintered hydroxyapatite	poly(L-lactide-co-glycolide) (PLGA) semi- crystalline poly(α-hydroxyester) co-polymer with CaCO_3_, with 45S5 Bioglass and with ICIE4 bioactive glass	Composite scaffolds	No information	Bioactive glass fillers were found to increase the polymer degradation and reduce polymer’s thermo-mechanical properties. Polymers with hydroxyapatite and CaCO_3_ are desirable polymer fillers.

**Table 5:** In vitro examination of materials—a mix of polylactide and bio-ceramics with additional material or factor.

Reference Number	Examined Material	Material Compared	Form of Applied Material	Type and Percentage of HAp/HAp in the Examined Material	Results Short
Adil Akkouch [67]	Mineralized type I collagen (Coll), hydroxyapatite (HAp), and poly(lactide-co-e-caprolactone) (PLCL)	PLCL scaffolds	3D scaffold with either a cylindrical (tube) structure or a cubic (cube) form, depending on the shape of the mould	Not specified	Thermal and mechanical evaluations proved, that the material is a resistant and elastic scaffold, able to promote osteoblast adhesion and proliferation.
Adil Akkouch [68]	tri-component osteogenic composite scaffold made of collagen (Coll), hydroxyapatite (HAp) and poly(L-lactide-co-caprolactone) (PLCL) cultured on human osteo- blast-like cells obtained by differentiation of dental pulp stem cells (DPSCs)	PLCL	Not mentioned	Not specified	The novel material Coll/HAp/PLCL yielded better results in each of the conducted studies: adhesion to DPSCs (grew faster), alkaline phosphatase activity, tissue mineralization (higher).
Ahmed Talal [24]	nHAp + PLA + Platelet Derived Growth Factors	PLA, tissue culture plastic	12 mm samples of composite films	10, 40, 70	Lowest percentage material had the highest osteoblasts proliferation rate. High concentration material had the highest ALP activity and was stated as a useful material for application a GTR membrane.
Ángel E. Mercado-Pagán [73]	4-arm poly(lactic acid urethane)-maleate (4PLAUMA) elastomer with nano-hydroxyapatite (nHA)	4PLAUMA	Composite samples	50% wt (1:1), 66,6% wt (1:2), 71% wt (2:5), 75% wt (1:3)	Stem and endothelial cells attachment properties of 4PLAUMA scaffold, as well as the compatibility, were stated to be proper. Material was voted as a great weight-bearing bone void filler.
Hae-Won Kim [74]	Bio-ceramic hydroxyapatite (HAp) in suspension in biopolymer poly(lactic acid) (PLA) mediated with HSA surfactant through the electrospinning process	PLA, PLA-HAp without HSA	Nanocomposite fiber	5 wt%	The HAp/PLA composite fiber proved to have superior osteoblastic cellular responses in comparison with PLA and a higher ALP activity. The use of HSA as a surfactant solved the problems associated with mixing bio-ceramics with biopolymers.
Jun-Sik Son [75]	HAp/PLA loaded with Dexamethasone	HAp scaffold	Scaffold	No information	The material of HAp and bio-ceramics mixed with DEX revealed increased ALP and protein levels in scaffold, and increased formation of calcified tissue compared to HAp only scaffold.
Jung Bok Lee [76]	PLLA cylinder with HAp and Simvastatin	No material compared	Composite microfibers and their cylinders	20.0%	Simvastatin promoted stimulation of bone formation. The PLLA cylinders display a potential to be included in 3D scaffold used in bone regeneration.
M. Mehdikhani-Nahrkhalaji [77]	Poly (lactide- co-glycolide)/bioactive glass/hydroxyapatite (PBGHA) nanocomposite	Titanium	10, 15 and 20 wt% nanoparticles of equal content of HAp and BG	No information	The 10 wt% nano particles were showed to provide the most desired coating for the material.
Marco C. Bottino [19]	neat PLCL + protein/lymer ternary blend + PLA:GEL + 10% n-HAp and PLA:GEL + 25% Metronidazole	Nothing	Periodontal membrane	10% wt	The method of fabrication enhanced predictability and durability of the material. The material is considered functional, with osteoconductive, inductive and antibacterial properties.

**Table 6:** Quality Assessment.

References	Material Used in the Study	Control Trial or a Material Compared	Content of HAp/FAp in the Material Expressed in Percentage Value	Total	Risk of Bias
Akiro Matsuo [20]	2	0	1	3	moderate
Akira Matsuo [3]	2	1	1	4	low
Andrea Vaz Braga Pintor [4]	3	0	1	4	low
Constantin A. Landes [14]	2	1	0	3	moderate
Hideo Shimizu [5]	2	0	1	3	moderate
Idalia A. W. Brito Siqueira [6]	2	1	0	3	moderate
In-Seok Song [21]	2	1	1	4	low
Jung Hyun Parl [17]	2	0	0	2	high
Koichiro Ueki [18]	2	1	0	3	moderate
K. Ueki [15]	2	1	1	4	low
Murat Cavit Cehreli [64]	2	1	ns	3	moderate
Osama Zakaria [7]	2	0	1	3	moderate
Ruggero Rodriguez y Baena [12]	3	1	0	4	low
Shintaro Sukegawa [25]	2	0	1	3	moderate
Shinya Tsumiyama [22]	2	0	0	2	high
Sun Jae Lee [23]	2	0	0	2	high
T Zislis [28]	3	1	0	4	low
Tohru Hayakawa [8]	2	1	1	4	low
Ueki Koichiro [16]	3	1	1	5	low
Vukoman Jokanović [9]	3	1	0	4	low
Y. Shikinami [26]	2	1	1	4	low
Akihiro Takayama [13]	1	1	1	3	moderate
Hao-Chieh Chang [27]	1	1	ns	2	high
Jung Bok Lee [10]	1	1	1	3	moderate
Miguel Noronha Oliveira [65]	1	1	ns	2	high
Mohamed H.Helal [11]	1	1	ns	2	high
Rung-Shu Chen [66]	1	1	ns	2	high
Bryan Taekyung Jung [69]	2	1	0	3	moderate
C Amnael Orozco-Díaz [29]	2	1	1	4	low
J.M. Taboas [70]	2	1	0	3	moderate
Kyung Mi Woo [71]	2	1	0	3	moderate
R.L. Simpson [72]	3	1	0	4	low
Adil Akkouch [67]	1	1	ns	2	high
Adil Akkouch [68]	1	1	ns	2	high
Ahmed Talal [24]	1	1	1	3	moderate
Ángel E. Mercado-Pagán [73]	1	1	1	3	moderate
Hae-Won Kim [74]	1	1	1	3	moderate
Jun-Sik Son [75]	1	1	0	2	high
Jung Bok Lee [76]	1	0	1	2	high
M. Mehdikhani-Nahrkhalaji [77]	1	1	0	2	high
Marco C. Bottino [19]	1	0	1	2	high

## Data Availability

Availability of supporting data—the datasets used and/or analyzed during the current study are available from the corresponding author on reasonable request.
